# ADMET-AI: a machine learning ADMET platform for evaluation of large-scale chemical libraries

**DOI:** 10.1093/bioinformatics/btae416

**Published:** 2024-06-24

**Authors:** Kyle Swanson, Parker Walther, Jeremy Leitz, Souhrid Mukherjee, Joseph C Wu, Rabindra V Shivnaraine, James Zou

**Affiliations:** Department of Computer Science, Stanford University, 353 Jane Stanford Way, Stanford, CA 94305, USA; Greenstone Biosciences, 3160 Porter Drive, Suite 140, Palo Alto, CA 94304, USA; Carleton College, One North College Street, Northfield, MN 55057, USA; Greenstone Biosciences, 3160 Porter Drive, Suite 140, Palo Alto, CA 94304, USA; Greenstone Biosciences, 3160 Porter Drive, Suite 140, Palo Alto, CA 94304, USA; Stanford Cardiovascular Institute, Stanford University, 265 Campus Drive, Stanford, CA 94305, USA; Greenstone Biosciences, 3160 Porter Drive, Suite 140, Palo Alto, CA 94304, USA; Department of Computer Science, Stanford University, 353 Jane Stanford Way, Stanford, CA 94305, USA; Department of Biomedical Data Science, Stanford University, 1265 Welch Road, Stanford, CA 94305, USA

## Abstract

**Motivation:**

The emergence of large chemical repositories and combinatorial chemical spaces, coupled with high-throughput docking and generative AI, have greatly expanded the chemical diversity of small molecules for drug discovery. Selecting compounds for experimental validation requires filtering these molecules based on favourable druglike properties, such as Absorption, Distribution, Metabolism, Excretion, and Toxicity (ADMET).

**Results:**

We developed ADMET-AI, a machine learning platform that provides fast and accurate ADMET predictions both as a website and as a Python package. ADMET-AI has the highest average rank on the TDC ADMET Leaderboard, and it is currently the fastest web-based ADMET predictor, with a 45% reduction in time compared to the next fastest public ADMET web server. ADMET-AI can also be run locally with predictions for one million molecules taking just 3.1 h.

**Availability and implementation:**

The ADMET-AI platform is freely available both as a web server at admet.ai.greenstonebio.com and as an open-source Python package for local batch prediction at github.com/swansonk14/admet_ai (also archived on Zenodo at doi.org/10.5281/zenodo.10372930). All data and models are archived on Zenodo at doi.org/10.5281/zenodo.10372418.

## 1 Introduction

The curation of large chemical repositories and advances in algorithms and computational hardware have significantly expanded the scale of *in silico* drug discovery campaigns. Structure-based virtual screening, pharmacophore modelling and, in particular, high-throughput molecular docking (HTMD) have been used to screen over a billion molecules against therapeutic targets of interest (Lyu *et al.* 2023). More recently, generative artificial intelligence (AI) approaches have been developed to design a vast array of compounds that are optimized for a particular therapeutic effect (Jacobs *et al.* 2021). Both HTMD and generative AI approaches yield a large number of potential hits with high efficacy, but many of these hits are not ideal for therapeutic development because they do not possess druglike properties (Bender *et al.* 2021). As a result, rapid and accurate screening of these hits for ideal druglike properties is critical for advancing molecules with higher probabilities of success (Feinberg *et al.* 2020). Specifically, for a small molecule to be at the centre of a therapeutic strategy and proceed from discovery to clinical trials, the compound must possess optimal Absorption, Distribution, Metabolism, Excretion, and Toxicity (ADMET) properties.

**Figure 1.**
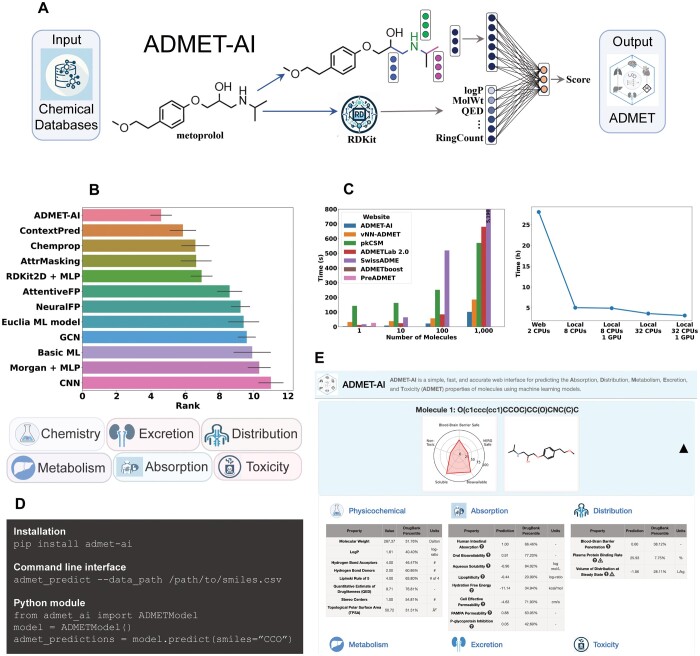
Overview of ADMET-AI. (A) An illustration of training an ADMET-AI graph neural network Chemprop-RDKit model. (B) The overall rank of ADMET-AI models on the Therapeutics Data Commons ADMET leaderboard of 22 ADMET datasets. Representative overall categories predicted by ADMET-AI are shown below. Error bars indicate standard error across datasets. (C) The computational efficiency of ADMET-AI. *Left panel*, the time (in seconds, median of three trials) for the ADMET-AI web server and other common ADMET web servers to make predictions on 1, 10, 100, or 1000 molecules from the DrugBank reference set. ADMETboost and PreADMET are limited to one molecule. Since pkCSM and SwissADME are limited to 100 and 200 molecules, respectively, their 1000 molecule times are computed as 100 molecule time ×10. *Right panel*, the time (in hours, median of three trials) for the ADMET-AI web server and various hardware configurations running the ADMET-AI local command line tool to make predictions on 1 million molecules from the DrugBank reference set (1000 copies of the 1000 molecule DrugBank set). Since the ADMET-AI web server is currently limited to 1000 molecules, its 1 million molecule time is computed as 1000 molecule time ×1000. (D) Commands needed to install and run the local version of ADMET-AI, either as a command line tool or as a Python module. (E) Predictions displayed on the ADMET-AI website (admet.ai.greenstonebio.com).

Here, we have developed ADMET-AI, a simple, fast, and accurate platform for ADMET property prediction that includes both a web interface and a Python package for local prediction (Fig. 1). ADMET-AI uses a graph neural network called Chemprop-RDKit (Fig. 1A), which was trained on 41 ADMET datasets from the Therapeutics Data Commons (TDC). ADMET-AI surpasses existing ADMET prediction tools in terms of speed and accuracy (Fig. 1B and C). Moreover, it provides additional useful features such as local batch prediction (Fig. 1D) and contextualized ADMET predictions using a reference set of approved drugs; these features are missing in most of the current ADMET prediction tools. We make ADMET-AI freely available and open-source as a resource to aid in the evaluation of large-scale chemical libraries for druglike compounds (Fig. 1E).

## 2 Model development

### 2.1 Data

ADMET-AI uses 41 ADMET datasets (10 regression, 31 classification) from the Therapeutics Data Commons (TDC, v0.4.1) (Huang et al., 2021b) . Of those 41 datasets, 22 datasets (9 regression, 13 classification) form the TDC ADMET Leaderboard (tdcommons.ai/benchmark/admet_group/overview), which enables a side-by-side comparison of ADMET-AI compared to other ADMET prediction models. In addition, from the 41 ADMET datasets, two multi-task datasets were created, one containing all 10 regression datasets and one containing all 31 classification datasets. This enables the training of just two models (one regression, one classification) that cover all 41 ADMET properties. Summary statistics for the ADMET datasets are listed in Supplementary Table S1, and further details on the preparation of the datasets are in the supplementary methods.

### 2.2 Model

ADMET-AI uses a deep learning model architecture for ADMET prediction called Chemprop-RDKit (available in Chemprop, v1.6.1) (Yang *et al.* 2019). Chemprop-RDKit consists of a graph neural network called Chemprop that is augmented with 200 physicochemical molecular features that are computed by the cheminformatics package RDKit (v2023.3.3) (rdkit.org). The Chemprop graph neural network computes simple features for each atom (e.g. atom type) and each bond (e.g. bond type) and then runs several steps of message passing with neural network layers to aggregate atom and bond information across the molecule to build a single representation of the whole molecule. This representation is then concatenated with the 200 RDKit features, and the combined representation is passed through several feed-forward neural network layers to predict one or more endpoints, depending on the dataset.

For each TDC dataset, one Chemprop-RDKit model was trained for each of the five train/validation/test splits, and the average performance across the five test splits for that dataset was recorded. At inference time, for each dataset, the average prediction is computed from an ensemble of models containing the five models trained on the five splits of that dataset. Ensembling is used since it can overcome incorrect biases in individual models and thereby improve the performance and robustness of the ensemble (Ali and Pazzani 1996).

### 2.3 Results

ADMET-AI has the best average rank among all models (Hu *et al.* 2019, Xiong *et al.* 2020, Huang *et al.* 2021a, Lee *et al.* 2021, Boral *et al.* 2022, Kipf and Welling 2017) that have been evaluated on the 22 datasets in the TDC ADMET Leaderboard (Fig. 1B, Supplementary Fig. S1). While other models are accurate on only a few ADMET properties, ADMET-AI is the best on average across all ADMET properties, thereby making it ideal for comprehensive ADMET analysis.

Across all 41 TDC ADMET datasets, ADMET-AI models trained on each dataset individually (‘single-task’) had overall strong performance, with an *R*^2^ >0.6 for five of the ten regression datasets and an AUROC (area under the receiver operating characteristic curve) >0.85 for 20 of the 31 classification datasets (Supplementary Fig. S2). ADMET-AI models trained on the two multi-task datasets achieved very similar performance (Supplementary Fig. S2). Notably, the two multi-task models (one for regression, one for classification) make predictions for all the properties significantly faster than the 41 single-task models. Therefore, the multi-task models were deployed in the ADMET-AI web server. The web server uses ensembles of the five models for each property to make predictions due to the benefit of ensembling (Supplementary Fig. S3).

Complete performance results are in Supplementary Table S1.

## 3 ADMET-AI web server

The ADMET-AI web server was built using Flask (v2.3.2) (flask.palletsprojects.com/en/2.3.x), which is a Python-based micro web framework. ADMET-AI possesses an intuitive user interface to display the predicted ADMET properties. In addition, each predicted ADMET value is contextualized via a comparison to a reference set of molecules from the DrugBank as described below. The ADMET-AI models can also be run locally for high-throughput ADMET prediction. Both as a web server and as a local package, ADMET-AI is significantly faster than publicly available ADMET prediction tools.

### 3.1 Drugbank reference

ADMET predictions can be difficult to interpret in isolation and can be better evaluated within the context of drugs with a similar therapeutic indication. As such, ADMET-AI incorporates a point of reference by comparing each query molecule to a set of approved molecules. This unique feature provides useful context for interpreting ADMET predictions and guides the user to the range of ADMET properties needed for their drug discovery project. ADMET-AI, therefore, curated a list of 2579 drugs from the DrugBank (v5.1.10) (Wishart *et al.* 2018) that have obtained regulatory approval as a reference set (Supplementary Table S2). The ADMET-AI multi-task models were applied to make predictions on these molecules for all ADMET endpoints. For any molecule queried on the ADMET-AI website, ADMET-AI computes the percentile of that molecule’s ADMET predictions relative to this DrugBank reference. Furthermore, since it is known that different classes of drugs have different ADMET requirements (e.g. acceptable toxicity for antineoplastics is much higher than that for antibiotics), the ADMET-AI website, therefore, allows the user to select an appropriate subset of the DrugBank reference based on Anatomical Therapeutic Chemical (ATC) codes (atcddd.fhi.no/atc/structure_and_principles) to use as the reference set for computing percentiles to add perspective during evaluation (common ATC codes are shown in Supplementary Fig. S4).

### 3.2 User experience

Users access the web interface of ADMET-AI at admet.ai.greenstonebio.com as shown in Fig. 1E. There are three options to input molecules: (i) by entering SMILES in a text box with each SMILES on a line, (ii) by uploading a CSV file containing multiple SMILES, or (iii) by drawing the structure of the molecule using an interactive tool that converts the drawing to a SMILES. Users can make predictions on up to 1000 molecules at a time. Next to the SMILES input, the user can create the DrugBank reference set by selecting an ATC code or by using the default of all DrugBank-approved molecules.

The 41 ADMET predictions, along with eight physicochemical properties computed by RDKit, are displayed on the website for up to 25 molecules (Fig. 1E), with predictions for the remaining molecules available for download. For each predicted molecule, a radar plot is shown as a quick summary of key components of druglikeness, including hERG toxicity (i.e. potential for cardiotoxicity and arrhythmias), blood–brain barrier (BBB) penetration (i.e. potential for CNS effects), solubility, oral bioavailability (i.e. ease of drug delivery), and potential for toxicity. The complete set of ADMET predictions for the molecule is shown in a set of tables, and each prediction is paired with the percentile of that prediction with respect to the DrugBank-approved reference set. For regression properties, the predicted values are displayed in the same units as in the dataset used to train the model [e.g. half life (*t*_1/2_) is predicted in terms of hours]. For binary classification properties, the predicted values are the probability that the molecule has the property (e.g. the probability of BBB penetration). For a simultaneous comparison of multiple molecules, ADMET-AI displays a summary scatter plot with two user-selected ADMET properties (one on each axis) as compared to the DrugBank reference set.

### 3.3 Local ADMET prediction

The ADMET-AI website enables fast and accurate ADMET prediction with no installation or computational experience required. ADMET-AI is also available as a Python package (github.com/swansonk14/admet_ai) for local ADMET prediction using the same multi-task ADMET models that power the ADMET-AI website. The single-task models are also available for download and use. The local version of ADMET-AI includes a command line tool called admet_predict that allows users to make predictions on massive datasets, such as those originating from large docking campaigns or generative AI models, that would exceed the computational capacity of the web server (Fig. 1D). The ADMET models can also be incorporated directly within a user’s local computational pipeline by importing the ADMET-AI models in just a few lines of Python code (Fig. 1D). The local version of ADMET-AI has no limit on the number of molecules that can be run through the model, and it automatically uses a GPU, if available, and multiple CPUs to increase prediction speed (Fig. 1C). The local version is also ideal for users who are privacy conscious and want to avoid putting their proprietary compounds through a web server, although it should be noted that the ADMET-AI web server does not store information on any molecules that are queried.

### 3.4 Comparison to alternatives: speed

While there are several publicly available ADMET prediction web servers currently available, ADMET-AI provides a powerful combination of accuracy, speed, and flexibility along with unique features such as the DrugBank reference set and local prediction. In addition, ADMET-AI is fully open source, making it easy to build upon and extend.

One of the most important qualities of an ADMET tool is the speed with which it can predict the ADMET properties of molecules. This is increasingly important as drug discovery projects rapidly grow in scale. To that end, the speed of ADMET-AI was compared to six other web servers that predict a wide range of ADMET properties: SwissADME (Daina *et al.* 2017), ADMETLab 2.0 (Xiong *et al.* 2021), pkCSM (Pires *et al.* 2015), vNN-ADMET (Schyman *et al.* 2017), ADMETboost (Tian *et al.* 2022), and PreADMET (Lee *et al.* 2003, Lee *et al.* 2004). Each server was used to make ADMET predictions for 1, 10, 100, or 1000 molecules from the DrugBank reference (Supplementary Table S3), and the median of three trials was recorded. ADMET-AI is the fastest publicly available web server for any number of molecules, with a 45% reduction in time over the next best server for 1000 molecules (Fig. 1C, left panel).

For truly large-scale analysis, local prediction is preferable to take advantage of greater CPU parallelism and GPU availability. To illustrate this, local versions of ADMET-AI with 8 or 32 CPU cores and with or without a GPU were used to make predictions on one million molecules from the DrugBank reference (1000 copies of the 1000 molecule DrugBank set). The local prediction speed was compared to the speed of the ADMET-AI web server, which uses two CPU cores and no GPU. Local ADMET-AI prediction with 32 CPU cores and a GPU reduces the ADMET prediction time from 28.1 h with the web server to 3.1 h locally, representing a further 89% reduction in time (Fig. 1C, right panel). Even a simple setup of 8 CPU cores and no GPU can make one million predictions in just 5 h. Therefore, ADMET-AI enables fast, large-scale ADMET prediction.

## 4 Conclusion

ADMET-AI is a simple, fast, and accurate platform for ADMET prediction. ADMET-AI uses a Chemprop-RDKit graph neural network for ADMET prediction that is currently the most accurate model on average across the TDC ADMET leaderboard. ADMET-AI is the fastest web-based ADMET predictor with a 45% reduction in time compared to the next fastest public ADMET web server. ADMET-AI uniquely provides context by comparing ADMET predictions on input molecules to predictions on approved drugs from the DrugBank, which can optionally be filtered to a specific drug category by ATC code. The ADMET-AI platform also includes a Python package with a command line tool for large-scale evaluation and a Python module for use within other drug discovery tools (e.g. generative AI models). ADMET-AI is an open-source, free, and easy-to-use platform that can serve as a powerful drug discovery tool for identifying compounds with favourable ADMET profiles for further development.

## Supplementary Material

btae416_Supplementary_Data

## Data Availability

The data underlying this article are available in Zenodo, at doi.org/10.5281/zenodo.10372418. Data was originally obtained from the Therapeutics Data Commons (v0.4.1) and DrugBank (v5.1.10) and then further processed for this study.
