# Supplementary material for: ADMET-AI: a machine learning ADMET platform for evaluation of large-scale chemical libraries
Source: Bioinformatics. 2024 Jun 24;40(7):btae416. doi: 10.1093/bioinformatics/btae416 (PMC11226862; doi:10.1093/bioinformatics/btae416)
Supplement: btae416_Supplementary_Data [file btae416_supplementary_data.zip › ADMET-AI Supplement.pdf]

Supplementary materials for ADMET-AI: A machine learning ADMET platform for evaluation of large-scale chemical libraries

Kyle Swanson[1,2,*], Parker Walther[3], Jeremy Leitz[2], Souhrid Mukherjee[2], Joseph C. Wu[4], Rabindra V. Shivnaraine[2,*], and James Zou[1,5,*]

[1]Department of Computer Science, Stanford University, [2]Greenstone Biosciences, [3]Carleton College, [4]Stanford Cardiovascular Institute, Stanford University, [5]Department of Biomedical Data Science, Stanford University

TDC Datasets

ADMET-AI models were trained on three subsets of the datasets from the Therapeutics Data Commons (TDC). Details of each subset are described below.

TDC Single-Task: The TDC Single-Task subset contains 41 ADMET datasets. It includes all ADMET datasets in the TDC except "hERG_Karim" and "herg_central," which are redundant with the "herg" dataset, and "ToxCast," which has 617 endpoints and is therefore challenging to interpret and to display on a web server. Among the 41 datasets, 10 are regression datasets and 31 are binary classification datasets. Each dataset was split into train, validation, and test sets using an 80%, 10%, 10% random split with five different random seeds. This makes it possible to train an ensemble of five models, each of which has seen a wide variety of molecular scaffolds, thereby improving generalisation of the models compared to using a scaffold split but at the potential cost of overly optimistic test set performance due to scaffold sharing between the train and test sets. For the regression datasets, the metrics were MAE (mean absolute error) and R^2 (coefficient of determination), and for the classification datasets, the metrics were AUROC (area under the receiver operating characteristic curve) and AUPRC (area under the precision-recall curve).

TDC Leaderboard: The TDC Leaderboard subset contains the 22 ADMET datasets in the TDC ADMET Benchmark Group (a subset of the 41 datasets in TDC Single-Task). Nine of the datasets are regression datasets and 13 of the datasets are binary classification datasets. The TDC maintains a leaderboard for the TDC Leaderboard datasets that ranks models according to their performance on a held-out test set for each dataset. This makes it possible to directly compare ADMET-AI's model against other state-of-the-art ADMET prediction tools. As per the TDC leaderboard guidelines, five models were trained, each one using a Murcko scaffold split with roughly 87.5% training data and 12.5% validation data created with different random seeds, and all models were evaluated using a single scaffold-split test set provided by the TDC. Scaffold splits represent the challenging but realistic scenario where the model is trained on molecules with certain chemical scaffolds but must generalise to new molecules with different scaffolds (e.g., new classes of drugs). For each dataset, the metric provided by the TDC is either mean absolute error (MAE) or Spearman's rank correlation coefficient (Spearman) for regression datasets and is either area under the receiver operating characteristic curve (AUROC) or area under the

precision-recall curve (AUPRC) for binary classification datasets. The leaderboard results reported here are as of October 4, 2023. Only models that are evaluated on all 22 datasets in the leaderboard are included.

TDC Multi-Task: The TDC Multi-Task subset contains two multi-task datasets built from TDC Single-Task. One dataset contains all 10 regression datasets (22,100 unique compounds), and the other contains all 31 binary classification datasets (35,774 unique compounds). The datasets were merged by matching SMILES so that any molecule that appears in multiple TDC Single-Task datasets appears as a single row in the multi-task dataset containing its SMILES and its endpoint values from every dataset it appears in. For datasets that the molecule does not appear in, the molecule's endpoint is filled in as a null value, and that endpoint is ignored during training and has no effect on the model's learning. The same random splitting scheme and metrics as for the TDC Single-Task datasets were used.

Supplementary Figure 1: TDC Leaderboard Results. Performance of ADMET-AI (red star) compared to other models (blue dots and crosses) on the TDC Leaderboard datasets according to four different metrics. Blue dots indicate models evaluated on all 22 TDC Leaderboard datasets while blue crosses indicate models evaluated on only some of the datasets (often just one). **A–B)** Performance on the regression datasets using either **(A)** Spearman rank correlation coefficient or **(B)** mean absolute error (MAE). **C–D)** Performance on the classification datasets using either **(C)** area under the receiver operating characteristic curve (AUROC) or **(D)** area under the precision-recall curve (AUPRC).

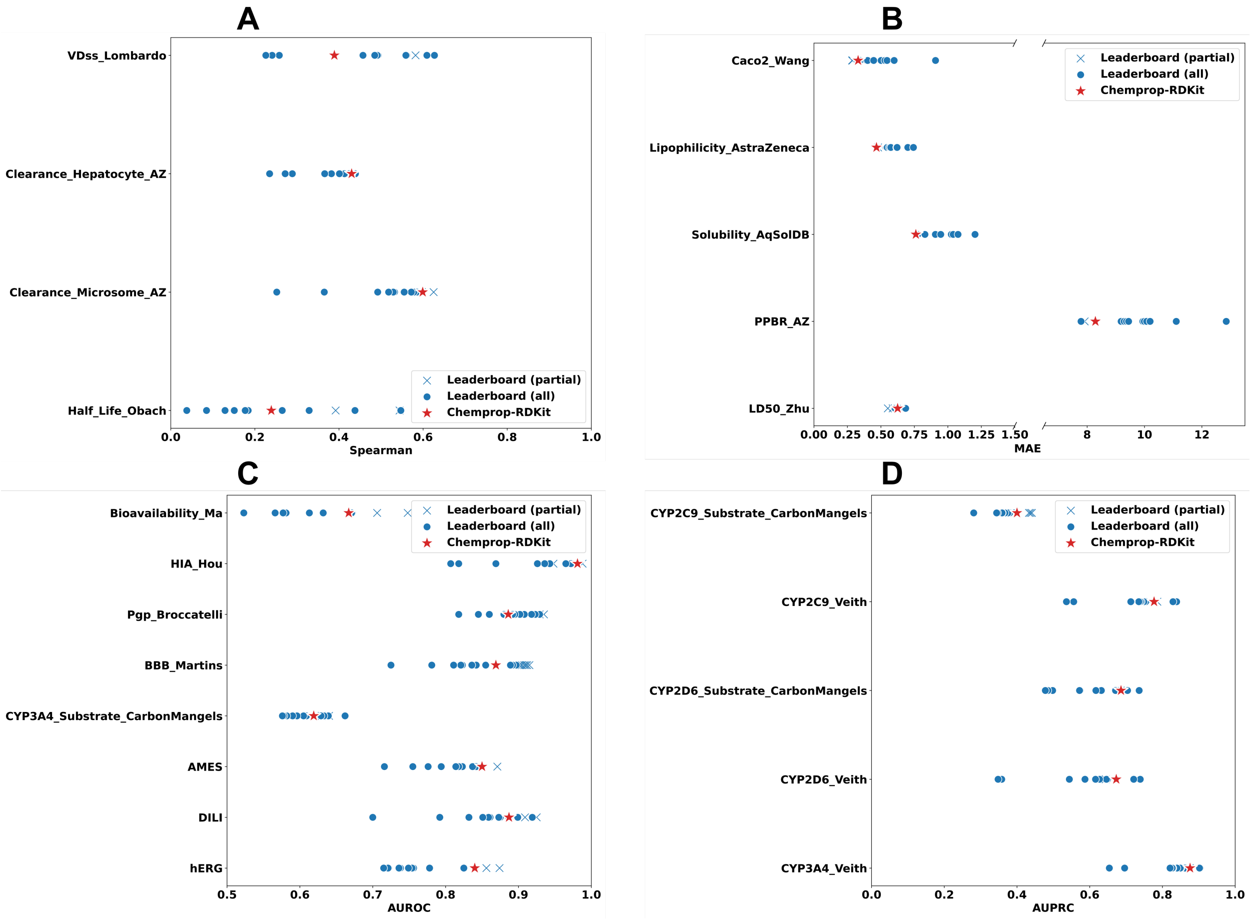

Supplementary Figure 2: TDC Single-Task vs TDC Multi-Task Results. Performance of ADMET-AI on the 41 TDC ADMET datasets, trained either as 41 single-task models or as two multi-task models, one for regression and one for classification. **A)** Performance on regression datasets using the coefficient of determination (R^2). **B)** Performance on regression datasets using mean absolute error (MAE). **C)** Performance on classification datasets using area under the receiver operating characteristic curve (AUROC). **D)** Performance on classification datasets using area under the precision-recall curve (AUPRC). Error bars indicate standard deviation across the five data splits.

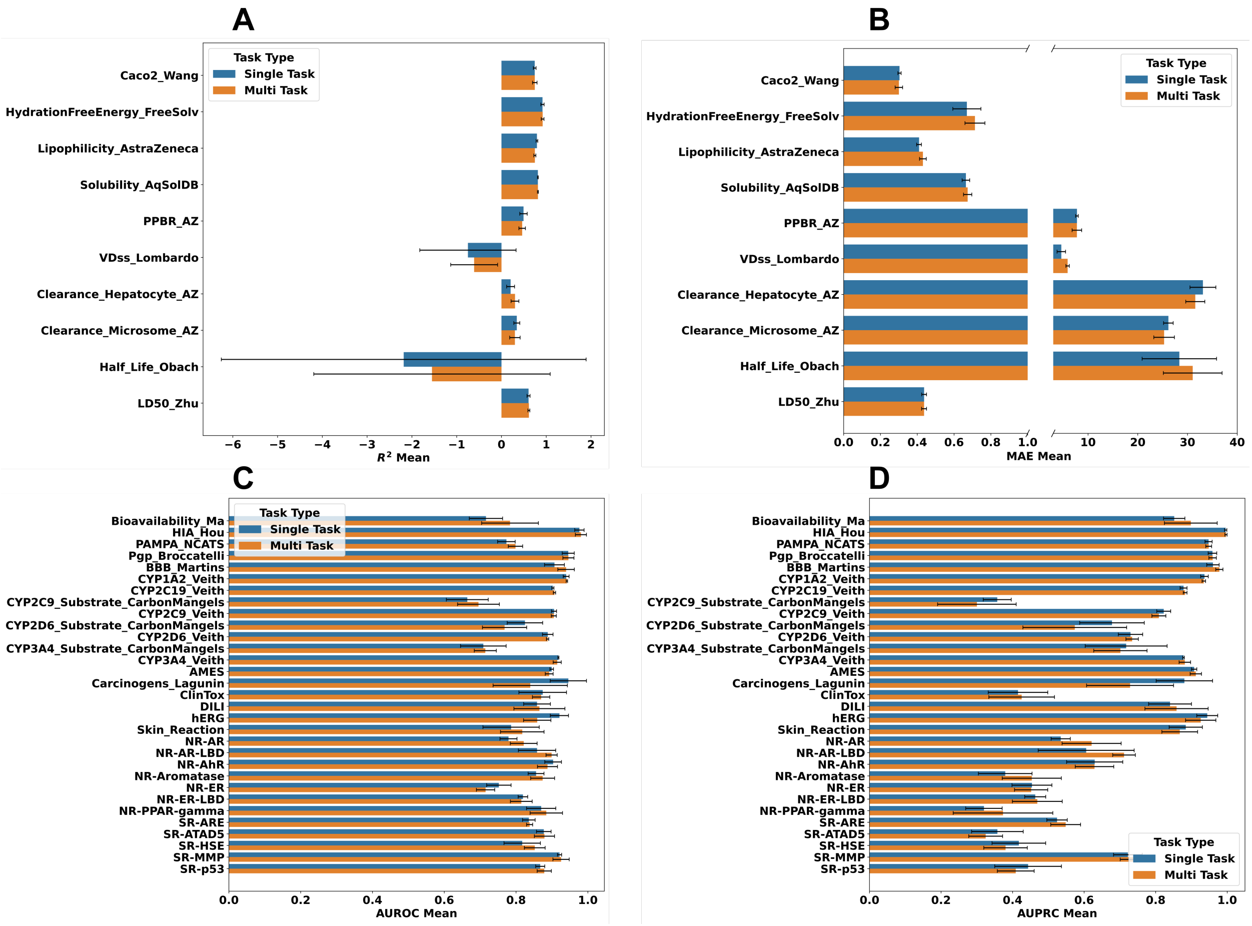

Supplementary Figure 3: Model Ensembling. Performance on the 22 TDC Leaderboard datasets using either an ensemble of five models or a single model (the first of the five models in the ensemble). **A–B)** Performance on the regression datasets using either **(A)** Spearman rank correlation coefficient or **(B)** mean absolute error (MAE). **C–D)** Performance on the classification datasets using either **(C)** area under the receiver operating characteristic curve (AUROC) or **(D)** area under the precision-recall curve (AUPRC).

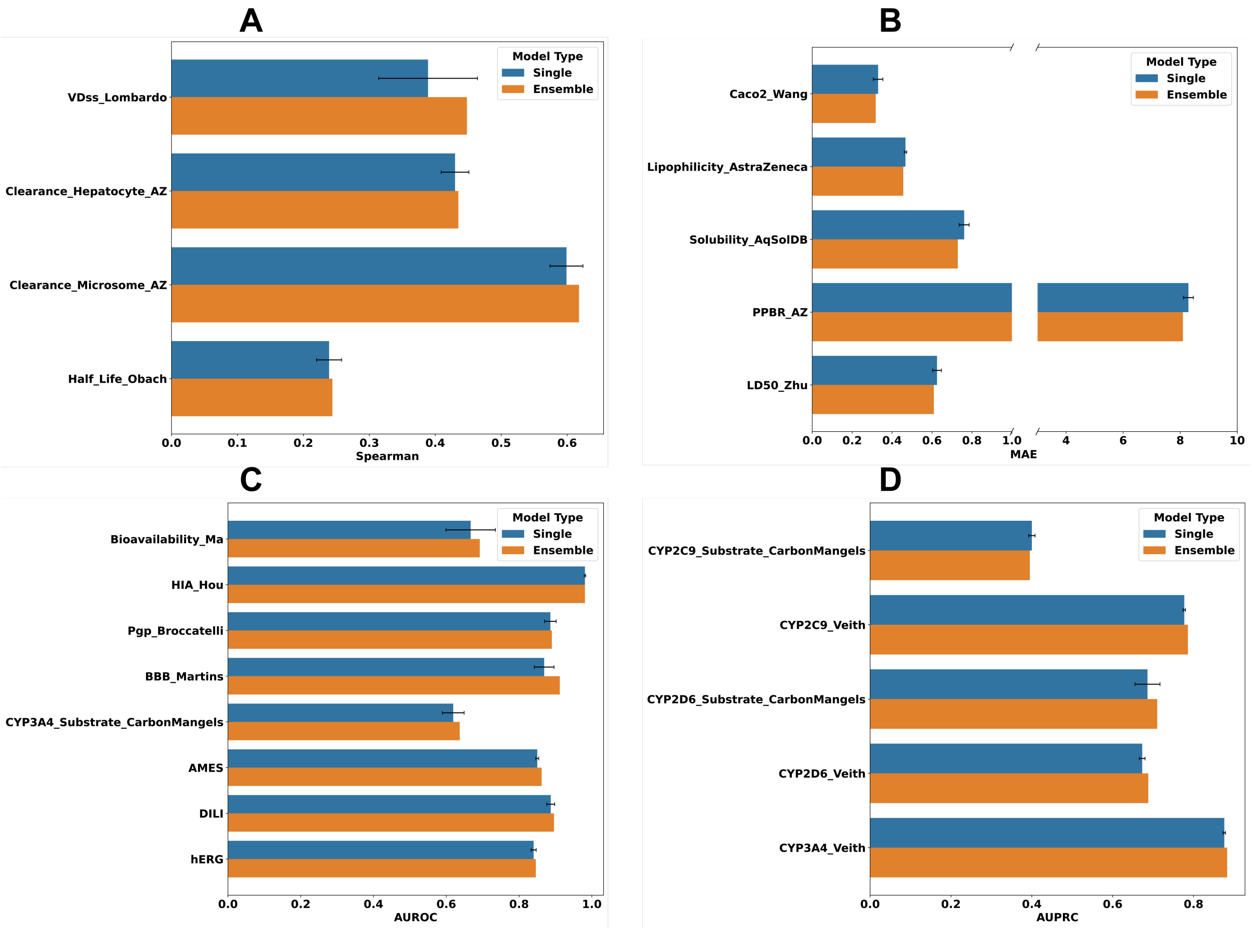

Supplementary Figure 4: DrugBank ATC Codes. Frequencies of Anatomical Therapeutic Chemical (ATC) codes in the 2,579 drugs in the DrugBank reference set at different ATC levels. **A)** The frequencies of the 14 ATC codes at level 1. **B)** The frequencies of the most common 25 of the 86 ATC codes at level 2. **C)** The frequencies of the most common 25 of the 223 ATC codes at level 3. **D)** The frequencies of the most common 25 of the 625 ATC codes at level 4.

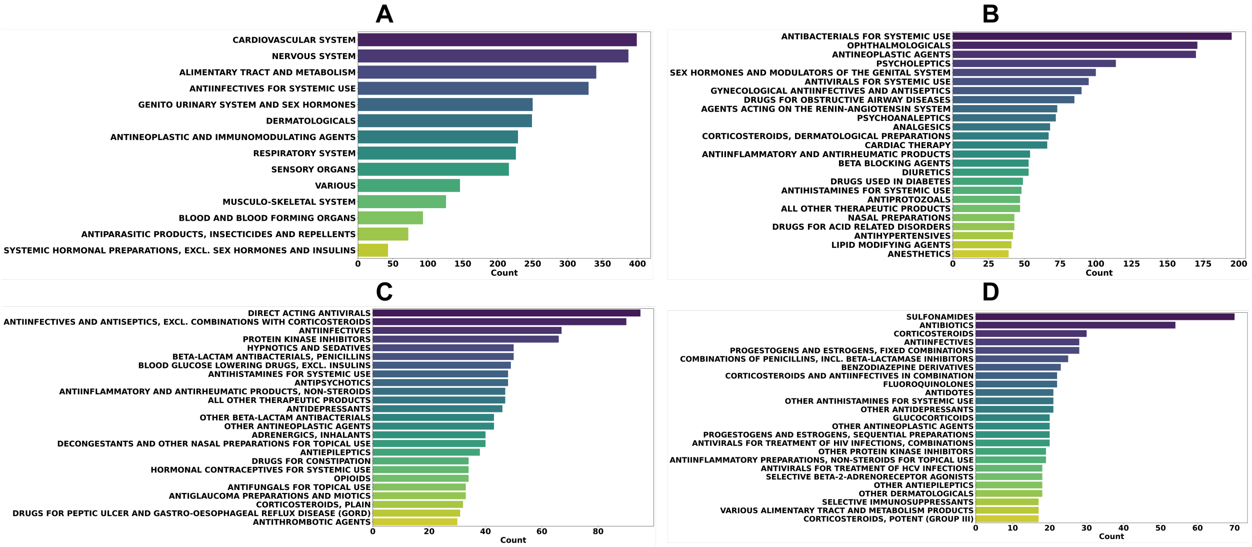

A
CARDIOVASCULAR SYSTEM
NERVOUS SYSTEM
ALIMENTARY TRACT AND METABOLISM
ANTIINFECTIVES FOR SYSTEMIC USE
GENITO URINARY SYSTEM AND SEX HORMONES
DERMATOLOGICALS
ANTINEOPLASTIC AND IMMUNOMODULATING AGENTS
RESPIRATORY SYSTEM
SENSORY ORGANS
VARIOUS
MUSCULO-SKELETAL SYSTEM
BLOOD AND BLOOD FORMING ORGANS
ANTIPARASITIC PRODUCTS, INSECTICIDES AND REPELLENTS
SYSTEMIC HORMONAL PREPARATIONS, EXCL. SEX HORMONES AND INSULINS
0 50 100 150 200 250 300 350 400
Count
B
ANTIBACTERIALS FOR SYSTEMIC USE
OPHTHALMOLOGICALS
ANTINEOPLASTIC AGENTS
PSYCHOLEPTICS
SEX HORMONES AND MODULATORS OF THE GENITAL SYSTEM
ANTIVIRALS FOR SYSTEMIC USE
GYNECOLOGICAL ANTIINFECTIVES AND ANTISEPTICS
DRUGS FOR OBSTRUCTIVE AIRWAY DISEASES
AGENTS ACTING ON THE RENIN-ANGIOTENSIN SYSTEM
PSYCHOANALEPTICS
ANALGESICS
CORTICOSTEROIDS, DERMATOLOGICAL PREPARATIONS
CARDIAC THERAPY
ANTIINFLAMMATORY AND ANTIRHEUMATIC PRODUCTS
BETA BLOCKING AGENTS
DIURETICS
DRUGS USED IN DIABETES
ANTIHISTAMINES FOR SYSTEMIC USE
ANTIPROTOZOALS
ALL OTHER THERAPEUTIC PRODUCTS
NASAL PREPARATIONS
DRUGS FOR ACID RELATED DISORDERS
ANTIHYPERTENSIVES
LIPID MODIFYING AGENTS
ANESTHETICS
0 25 50 75 100 125 150 175 200
Count
C
DIRECT ACTING ANTIVIRALS
ANTIINFECTIVES AND ANTISEPTICS, EXCL. COMBINATIONS WITH CORTICOSTEROIDS
ANTIINFECTIVES
PROTEIN KINASE INHIBITORS
HYPNOTICS AND SEDATIVES
BETA-LACTAM ANTIBACTERIALS, PENICILLINS
BLOOD GLUCOSE LOWERING DRUGS, EXCL. INSULINS
ANTIHISTAMINES FOR SYSTEMIC USE
ANTIPSYCHOTICS
ANTIINFLAMMATORY AND ANTIRHEUMATIC PRODUCTS, NON-STEROIDS
ALL OTHER THERAPEUTIC PRODUCTS
ANTIDEPRESSANTS
OTHER BETA-LACTAM ANTIBACTERIALS
OTHER ANTINEOPLASTIC AGENTS
ADRENERGICS, INHALANTS
DECONGESTANTS AND OTHER NASAL PREPARATIONS FOR TOPICAL USE
ANTIEPILEPTICS
DRUGS FOR CONSTIPATION
HORMONAL CONTRACEPTIVES FOR SYSTEMIC USE
OPIOIDS
ANTIFUNGALS FOR TOPICAL USE
ANTIGLAUCOMA PREPARATIONS AND MIOTICS
CORTICOSTEROIDS, PLAIN
DRUGS FOR PEPTIC ULCER AND GASTRO-OESOPHAGEAL REFLUX DISEASE (GORD)
ANTITHROMBOTIC AGENTS
0 20 40 60 80
Count
D
SULFONAMIDES
ANTIBIOTICS
CORTICOSTEROIDS
ANTIINFECTIVES
PROGESTOGENS AND ESTROGENS, FIXED COMBINATIONS
COMBINATIONS OF PENICILLINS, INCL. BETA-LACTAMASE INHIBITORS
BENZODIAZEPINE DERIVATIVES
CORTICOSTEROIDS AND ANTIINFECTIVES IN COMBINATION
FLUOROQUINOLONES
ANTIDOTES
OTHER ANTIHISTAMINES FOR SYSTEMIC USE
OTHER ANTIDEPRESSANTS
GLUCOCORTICOIDS
OTHER ANTINEOPLASTIC AGENTS
PROGESTOGENS AND ESTROGENS, SEQUENTIAL PREPARATIONS
ANTIVIRALS FOR TREATMENT OF HIV INFECTIONS, COMBINATIONS
OTHER PROTEIN KINASE INHIBITORS
ANTIINFLAMMATORY PREPARATIONS, NON-STEROIDS FOR TOPICAL USE
ANTIVIRALS FOR TREATMENT OF HCV INFECTIONS
SELECTIVE BETA-2-ADRENORECEPTOR AGONISTS
OTHER ANTIEPILEPTICS
OTHER DERMATOLOGICALS
SELECTIVE IMMUNOSUPPRESSANTS
VARIOUS ALIMENTARY TRACT AND METABOLISM PRODUCTS
CORTICOSTEROIDS, POTENT (GROUP III)
0 10 20 30 40 50 60 70
Count
